# Direct high-altitude observations of 2-methyltetrols in the gas- and particle phase in air masses from Amazonia

**DOI:** 10.1039/d4fd00179f

**Published:** 2025-02-28

**Authors:** Claudia Mohr, Joel A. Thornton, Manish Shrivastava, Anouck Chassaing, Ilona Riipinen, Federico Bianchi, Marcos Andrade, Cheng Wu

**Affiliations:** a Paul Scherrer Institute (PSI) Switzerland; b University of Washington USA; c Pacific Northwest National Laboratory USA; d Stockholm University Sweden; e University of Helsinki Finland; f Universidad Mayor de San Andres Bolivia; g University of Gothenburg Sweden cheng.wu@gu.se; h ETH Zurich Switzerland; i Bolin Centre for Climate Research Sweden

## Abstract

We present direct observations of 2-methyltetrol (C_5_H_12_O_4_) in the gas- and particle phase from the deployment of a Filter Inlet for Gases and Aerosols coupled to a Time-of-Flight Chemical Ionization Mass Spectrometer (FIGAERO-CIMS) during the Southern Hemisphere High Altitude Experiment on Particle Nucleation and Growth (SALTENA), which took place between December 2017 and June 2018 at the high-altitude Global Atmosphere Watch station Chacaltaya (CHC) located at 5240 m a s l in the Bolivian Andes. 2-Methyltetrol signals were dominant in a factor resulting from Positive Matrix Factorization (PMF) identified as influenced by Amazon emissions. We combine these observations with investigations of isoprene oxidation chemistry and uptake in an isolated deep convective cloud in the Amazon using a photochemical box model with coupled cloud microphysics and show that, likely, 2-methyltetrol is taken up by hydrometeors or formed *in situ* in the convective cloud, and then transported in the particle phase in the cold environment of the Amazon outflow and to the station, where it partially evaporates.

## Abstract 2

We present direct observations of 2-methyltetrol in the gas- and particle phase from the high-altitude measurement station Chacaltaya in the Bolivian Andes and investigate its sources and transport in air masses from Amazonia.

## Graphical abstract

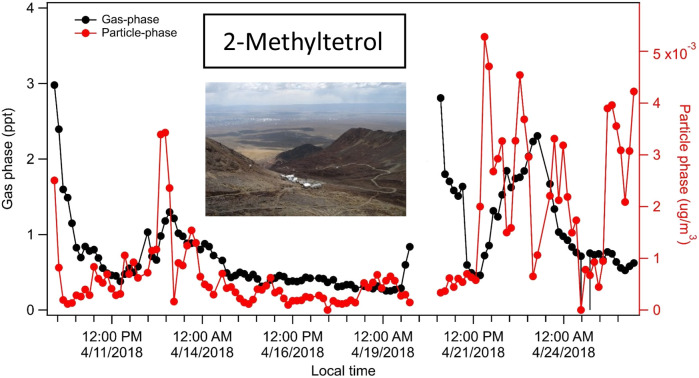

## Introduction

Chemical processes in the free troposphere (here operationally defined as the region >5000 m a s l) take place in a cold climate where temperatures ranging from close to 0 °C to ∼−80 °C affect the kinetics and thermodynamics of reactions and phase changes. The presence of chemical constituents is determined by solar radiation, chemical processes, mixing and transport,^1^ direct emissions are scarce. Convective transport brings air masses with higher concentrations of particles and trace gases from the lower parts including the planetary boundary layer (PBL) to the higher-altitude regions of the troposphere. Cloud processing is a relevant factor for the chemistry of the free troposphere.^2^ The chemical reactions in the cold environment of the high-altitude regions have climate and air quality impacts on the regional to hemispheric scale due to the longer residence time and thus farther transport of constituents.

An example of the synergistic effects of low temperature, convective transport and photochemistry of organic compounds emitted in the PBL is new particle formation (NPF) in the tropical upper troposphere above Amazonia.^3^ The Amazon stores 50% of tropical-forest carbon^4^ and is one of the major source regions of atmospheric volatile organic compounds (VOCs). VOCs are produced by plants for *e.g.* protection and communication.^5^ Globally, vegetation emits on the order of 1 Gt C per year in the form of VOCs,^6,7^ with isoprene (C_5_H_8_) as the dominating species, especially in the tropics. An estimated ∼100 Tg of isoprene are emitted by the Amazon per year.^6^ Further VOC emissions in the Amazon include other terpenoids such as monoterpenes, sesquiterpenes and diterpenes, methanol and other oxygenated compounds.^8^ Accordingly, the major fraction of the organic aerosol (OA) loading over the Amazon basin is biogenic in nature,^9,10^ with anthropogenic perturbations from urban areas and biomass burning,^11^ especially in the dry season.

Upon emission, these reactive compounds get oxidized, forming oxygenated VOCs (OVOCs) that due to their lower volatility compared to their precursors can partition into the particle phase to form secondary organic aerosol (SOA). A major pathway for isoprene to form SOA is *via* oxidation by hydroxyl radicals (OH) producing organic peroxy radicals, which, under conditions with low NO_*x*_, react with hydroperoxyl radicals to form isoprene hydroxy hydroperoxides (ISOPOOH).^12^ Where NO is present, its reaction with the peroxy radicals eventually leads to the formation of organonitrates and other nitrogen-containing compounds.^13^ ISOPOOH further reacts with OH leading to the formation of isoprene epoxydiols (IEPOX). Reactive uptake of IEPOX results in the acid-catalyzed formation of 2-methyltetrols.^14^ Simulations indicate IEPOX-SOA makes up 23% of surface concentrations of OA measured in the Amazon in the month of March.^10^

OVOCs are generally thought of and consequently simulated as chemically formed *in situ* in the atmosphere, however, recent findings show that OVOCs can also be biologically produced by plants as metabolic by-products or intermediates of biochemical processes and directly released into the atmosphere.^15^ One such example are 2-methyltetrols (C_5_H_12_O_4_), with some of the isomers being isoprene metabolism byproducts in plants,^16^ and others being produced in the atmosphere from isoprene oxidation.^17,18^ Direct near-canopy observations of 2-methyltetrols in the central Amazon could only be explained by direct emissions from the forest.^15^

Whereas the oxidation products of isoprene and directly emitted OVOCs contribute to SOA mass, they are in general too volatile for NPF in the Amazon PBL. However, in the cold environment of the upper free troposphere above Amazonia (temperature of about −50 to −60 °C at an altitude of ∼12 km), their apparent volatility is low enough to play a driving role in NPF.^3^ Earlier airborne observations have shown aerosol particle number concentrations in the tropical upper troposphere over the Amazon region at altitudes between 8 and 15 km that exceed concentrations in the PBL by 1–2 orders of magnitude, with the mass dominated by organic compounds and largely deprived of particulate species directly emitted in the PBL (*e.g.* black carbon).^19^

These earlier observations resulted in a conceptual model^19^ where during cloud updrafts in deep convective systems, pre-existing particles get scavenged, while more volatile VOCs such as isoprene and OVOCs emitted from the Amazon get transported to higher altitudes. There, photooxidation in the presence of NO_*x*_ from lightning leads to their oxidation to less volatile compounds, and supported by the low temperature, nucleation of new particles.^20–22^ Bardakov *et al.*^23^ have recently presented a photochemical box model with coupled cloud microphysics along individual air parcel trajectories extracted from simulations of a deep convective cloud using the large eddy simulation solver MIMICA^24^ to study the transport of gases related to isoprene photooxidation in a convective cloud. According to their simulations, up to ∼30% of the isoprene emitted in the PBL could be transported by an ‘average’ parcel into the cloud outflow (for a system with no lightning and efficient gas condensation on ice). Zha *et al.*^25^ directly observed nitrogen-containing isoprene oxidation products in free troposphere air masses from the Amazon at the Global Atmosphere Watch (GAW) research station Chacaltaya (CHC) in the Bolivian Andes at 5240 m a s l, providing first observational evidence for the conceptual model. Recent direct measurements above the Amazon together with laboratory studies have now confirmed these earlier results.^3,26^

Whereas these efforts have shed light on NPF mechanisms in the upper troposphere above Amazon, formation pathways of SOA and the role of isoprene photooxidation remain to be fully constrained in this environment. Shrivastava *et al.*^27^ have found that they need to evoke transport of directly emitted 2-methyltetrols to the upper troposphere and subsequent condensation onto pre-existing particles to explain IEPOX-SOA fractions in OA observed at 12–14 km altitude above the Amazon, as the presumably glassy state of the high-altitude OA due to low temperature and humidity would inhibit reactive uptake of IEPOX. Direct observations have remained scarce.

Here we show direct measurements of both gas- and particle-phase 2-methyltetrols in air masses arriving from the Amazon basin to the GAW research station CHC in the Bolivian Andes. We use these measurements, together with a chemical box model coupled with cloud microphysics, to investigate potential source mechanisms for the 2-methyltetrols. Our study underlines the regional extent of Amazon emissions and their chemistry in the low-temperature environment of the free troposphere.

## Results and discussion

### Observations of C_5_H_12_O_4_ in the gas- and particle phase at the high-altitude research station Chacaltaya in the Bolivian Andes

We deployed a Filter Inlet for Gases and Aerosols coupled to a Time-of-Flight Chemical Ionization Mass Spectrometer (FIGAERO-CIMS,^28,29^ Experimental) at the Global Atmosphere Watch (GAW) research station Chacaltaya^30^ (CHC) at 5240 m a s l in the Bolivian Andes. These measurements were part of the Southern Hemisphere High Altitude Experiment on Particle Nucleation and Growth (SALTENA,^31^ Experimental) campaign taking place between December 2017 and June 2018. The aim of this measurement campaign was to identify sources, formation mechanisms, transport, and characteristics of aerosol populations in this largely undersampled region. The mean temperature at CHC during the measurements was −0.2 ± 2 °C. The FIGAERO-CIMS was fully operational during April and May, *i.e.* the transition period between the wet and the dry season. Particle-phase compounds in the FIGAERO-CIMS are analyzed *via* desorption of particles collected on an inline particle filter using a controlled temperature ramp, yielding a thermogram (signal as a function of desorption temperature) which gets integrated for total particle-phase signal.

**Fig. 1:**
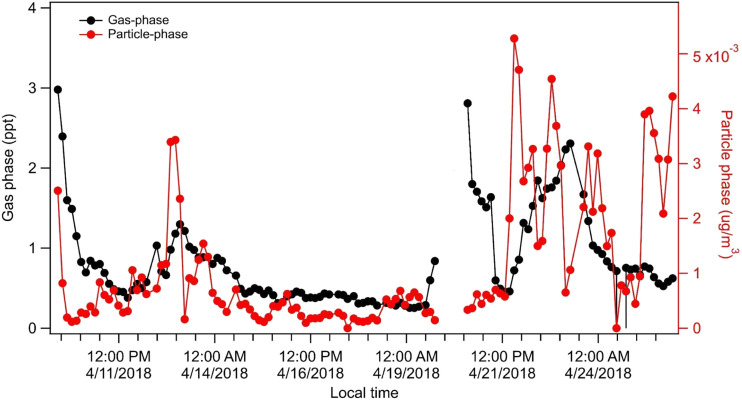
Time series of 2-methyltetrol (C_5_H_12_O_4_, MTeth) in gas and particle phase observed using a FIGAERO-CIMS at the GAW station CHC during the transition season in 2018.

The FIGAERO-CIMS captured a significant signal of C_5_H_12_O_4_, 2-methyltetrol (MTeth), in both the gas- and particle phase (Fig. 1). Gas-phase concentrations were around half a ppt in early April and increased to above 1 ppt in the second part of the month. Particle-phase concentrations were below 1 × 10^−3^ μg m^−3^ in early April and increased to ∼4 × 10^−3^ μg m^−3^ towards the end of the month (for estimates of sensitivity see Experimental). During the transition season in 2018, CHC was largely influenced by air masses transported in the free troposphere from the Amazon region during 9th–13th and 21st–26th of April. Both these periods coincide with higher concentrations of MTeth in the gas- and particle phases (Fig. 1).

### Positive matrix factorization of the particle-phase data reveals a factor representative of Amazon air, dominated by C_5_H_12_O_4_

We performed positive matrix factorization (PMF) on the particle-phase data of the FIGAERO-CIMS using the approach described by Buchholz *et al.*^32^ and in the Experimental in more detail. Briefly, the time series of the thermal desorption profiles of the particulate compounds measured by FIGAERO-CIMS were analyzed with PMF to identify source factors in both the chemical and volatility space, as well as their contributions over time (factor time series). By performing FIGAERO-PMF on the time series of about 1000 particle-phase organic species for two months (April and May 2018), five PMF factors were identified, which represent the chemical composition of particles in the air masses from different source regions. The time series of a PMF factor identified as representative of organic aerosol from species emitted by the Amazon is highly correlated with the time series of air masses originating from the Amazon region (details see Experimental).

**Fig. 2:**
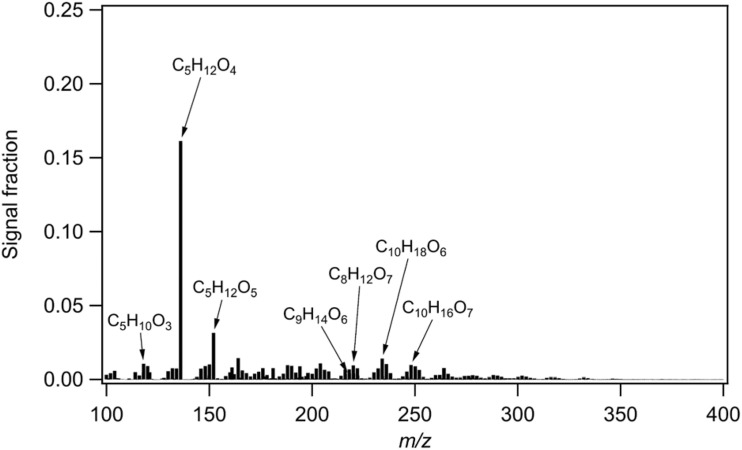
Mass spectrum of the Amazon rainforest factor identified *via* positive matrix factorization (PMF) of the particle-phase FIGAERO-CIMS data.

MTeth is the dominating compound of the Amazon PMF factor (Fig. 2), with contributions of 16% to the total signal and 12% to the total mass of this factor. The average thermogram shape of MTeth is mainly monomodal with a maximum desorption temperature (*T*_max_) of about 60–80 °C, which is similar to that of a 2-methyltetrol standard thermogram analyzed by D'Ambro *et al.*,^14^ which also has a monomodal shape with a *T*_max_ of about 60 °C. The other major species of the Amazon factor (Fig. 2) include C_5_H_12_O_5_ and C_5_H_10_O_3_ (likely alkene triols). There are also a few prominent C8–10 compounds, C_8_H_12_O_6,7_, C_9_H_14_O_6_, C_10_H_18_O_6_, C_10_H_16_O_7_, likely from monoterpene oxidation. The mass fraction of MTeth we measured is comparable to that observed during aircraft measurements over the Amazon rainforest (close to the source): Schulz *et al.*^33^ reported IEPOX-SOA fractions of OA mass concentrations of 23% near the surface and of 9% in the upper troposphere. MTeth is a commonly measured species from IEPOX-SOA^12,17^ and was first observed in ambient air in aerosols from the Amazonian rainforest.^34^ It can be explained by OH radical-initiated photooxidation of isoprene under low NO_*x*_ conditions.

We further compared the Amazon rainforest factor mass spectrum with that from both laboratory experiments of isoprene oxidation^35^ and field observations^36,37^ using the same instrument, *i.e.* a chemical ionization mass spectrometer using iodide as a reagent ion. While MTeth is an early-generation product of isoprene oxidation, both laboratory and field mass spectra of isoprene SOA are dominated by C5 compounds with 5–6 oxygen atoms, which have a higher degree of oxygenation compared to the compounds dominating the signal in our Amazon rainforest mass spectrum. Such differences indicate potentially different sources and/or oxidation mechanisms. It has been suggested recently that direct emissions of MTeth gases formed by in-plant biochemical oxidation and/or oxidation of deposited IEPOX gases on the surface of soils and leaves could play an important role in IEPOX-SOA in the upper troposphere (UT) above the Amazon basin.^27^

### Investigation of transport of C_5_H_12_O_4_ through convective clouds

**Fig. 3:**
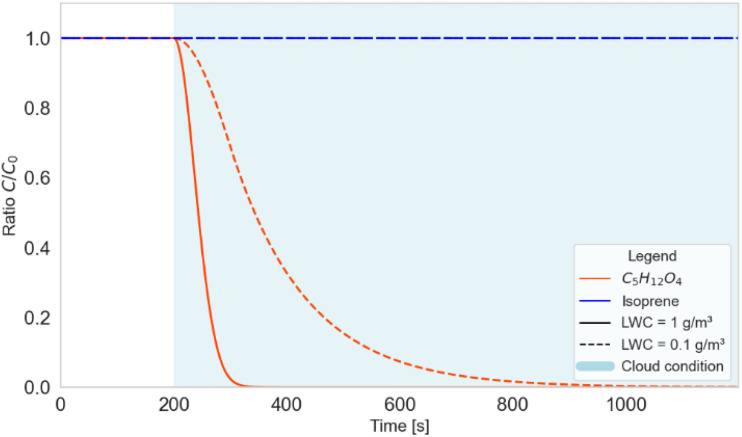
Simulation of expected transport of isoprene and MTeth through a convective cloud for a thin and a thick cloud case. The decrease of signal is due to uptake of MTeth by hydrometeors.

In order to check this hypothesis, we investigated the transport of MTeth through deep convective clouds by using CloudChem, a chemical box model coupled with cloud microphysics written in Python^23,24^ to evaluate the uptake of isoprene and MTeth by water and ice hydrometeors. Henry's law constants, characterising the solubility of a compound, were taken from Sander.^38^ Here, we simulated an air parcel starting from an initial concentration of isoprene and MTeth and a cloud-free condition. At *t* = 200 s, water droplets were introduced into the system. To investigate the sensitivity to cloud parameters, two cases were simulated: a thick cloud (Liquid Water Content (LWC) = 1 g m^−3^) and a thin cloud (LWC = 0.1 g m^−3^). The simulation used fixed atmospheric conditions at the surface (*T* = 298 K and *P* = 1013.25 mbar) and the chemistry was shut down. As can be observed in Fig. 3, for both cloud cases, a thick and a thin cloud, C_5_H_12_O_4_ does not survive the transport in the presence of water droplets and will be taken up by the hydrometeors. This is in stark contrast to isoprene, which does not show a reduction in concentration as the cloud is introduced to the system. We note here that temperature is kept constant across the entire simulation and thus the uptake of C_5_H_12_O_4_ by hydrometeors represents a lower limit. From these results we conclude that MTeth is likely not transported as a gas to the UT in deep convective clouds without being taken up by the cloud droplets. Isoprene on the other hand serves as an example of a gas that can be transported to the upper troposphere because of its extremely low solubility.

These results indicate that the MTeth we observed with the FIGAERO-CIMS at CHC in the gas- and particle phase in high-altitude air masses from the Amazon is likely not the result of condensation of MTeth emissions from the Amazon transported to the upper troposphere *via* deep convection and condensing onto pre-existing particles in the colder environment. We rather speculate that the MTeth, which is being directly emitted by the vegetation in the Amazon, is taken up by hydrometeors in the convective cloud system, and then released in the outflow of the Amazon as cloud droplets evaporate or upon freezing, similar to what was described for ammonia (NH_3_) by Wang *et al.*^39^

### Transport of air masses to CHC

We observed both gas- and particle-phase MTeth in the air masses transported from the Amazon region. On average about 24% of the air arriving at CHC originated from the boundary layer, and the other 76% came from the colder environment of the free troposphere (FT).^34^ In order to compare the near-surface transport and transport *via* free troposphere, we plot the particle-phase mass fraction (*P*/(*P* + *G*), *P* = particle-phase mass, *G* = gas-phase mass) and total amount from both gas- and particle phases as a function of the fraction of free troposphere air mass to the total air mass.

**Fig. 4:**
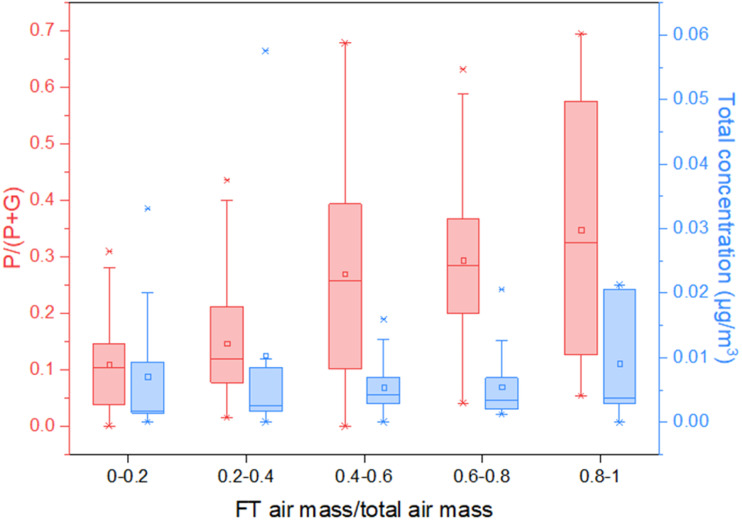
Particle-phase mass fraction (*P*/(*P* + *G*)) and the total mass of both gas- and particle phases of MTeth as a function of the contribution of the free troposphere (FT) air mass to the total air mass.

As Fig. 4 shows, when the air mass was mainly from the surface, *i.e.* the fraction of air mass from the FT low, the average particle fraction of MTeth was about 10%. When more FT air mass contributed to the total air mass measured, a much higher MTeth particle fraction was detected, with an average value of above 30%. Although the particle fraction detected by the FIGAERO-CIMS may have uncertainties, the trend clearly shows the higher particle fraction ratios in the FT air mass than that in the surface air mass. It is also worth pointing out that with increasing contribution of FT air mass, the total amount of MTeth did not significantly decrease, although a few data points with total mass higher than 0.03 μg m^−3^ were observed for an FT fraction lower than 40%. This indicates effective transport and/or formation of MTeth in the upper troposphere.

Shrivastava *et al.*^27^ proposed the condensation of MTeth gases onto pre-existing particles as the major path for its transport. With their regional model, more than 99% of the gases partitioned to the particle-phase OA in the upper troposphere due to extremely low temperatures, while with warmer temperatures near the surface, a smaller fraction (50–70%) of these gases exist in the particle phase. Our observations of higher particle fractions in the FT air mass are partly consistent with their study. However, due to the different spatial scales and constraints for transport and wet removal, a direct comparison is not meaningful.

## Experimental

### Southern Hemisphere High Altitude Experiment on Particle Nucleation and Growth (SALTEÑA)

The SALTEÑA campaign^31^ was conducted at the Global Atmosphere Watch (GAW) Chacaltaya mountain station (5240 m a s l; 16.35° S, 68.13° W) in Bolivia. Chacaltaya is a mountain with a horizon open to the south and west, facing the Altiplano, which is a high plateau at 3800 m a s l. Behind Chacaltaya, to the north and east, the high peaks of the Cordillera Real mountain range separate it from the Amazon Basin. This high-altitude site is influenced by both local and regional sources. The latter consists of long-range Amazon rainforest transport. In this study, we focus on the transition season, namely April in 2018 (10th–26th of April), when the station was greatly influenced by air masses from the Amazon region.

### FIGAERO-CIMS

A Filter Inlet for Gases and Aerosols coupled to a Time-of-Flight Chemical Ionization Mass Spectrometer (FIGAERO-CIMS) was utilized to measure the chemical composition of organic aerosols. The design and operation of the FIGAERO-CIMS were similar to those described in previous studies.^28,29,40^ In this study, the FIGAERO inlet was coupled to a high-resolution time-of-flight chemical-ionization mass spectrometer (HR-ToF-CIMS) (*M*/Δ*M* ∼ 5000–6000), and I^−^ was used as reagent ion. A corona source and an X-ray generator were used to ionize methyl iodide and produce the reagent ion in a nitrogen flow in April and May, respectively. The changes in the sensitivity due to low ambient pressures at high altitude and different settings with these two sources were considered. Particles were collected on a 25 mm Zefluor® PTFE filter (Pall Corp.) inside the FIGAERO *via* a sampling port (stainless steel tube of *ca.* 1.5 m length, inner diameter (ID) = 6 mm, flow rate 5 L min^−1^). The duration of particle-phase sampling was 120 min. Particle blanks were performed every few days. During particle-phase collection, gases were measured *via* an ID = 6 mm Teflon tube of ∼1 m length at 5 L min^−1^.

When the particle-phase sampling was completed, the gas-phase measurement was switched off and particles on the filter were desorbed by a flow of ultra-high-purity (99.999%) nitrogen. A FIGAERO desorption round lasted 55 min: 20 min of ramping the temperature of the nitrogen flow from ambient temperature up to 200 °C were followed by a 20 min “soak period” at a constant temperature of 200 °C, and 15 min of cooling down to room temperature. The mass spectral signal evolutions as a function of desorption temperature are termed thermograms.^28^ The integration of thermograms of individual compounds (cooling period excluded) yields their total signal in counts per deposition. Here we convert the counts per deposition into concentrations and the sensitivity calculations due to different settings are discussed in detail in Heitto *et al.*^41^

### Positive matrix factorization (PMF)

Positive matrix factorization (PMF) was performed using FIGAERO particle-phase data, specifically the time series of thermograms for individual compounds.^32^ This approach incorporates an additional dimension of volatility. The input data comprised desorption profiles from 278 filters collected using the FIGAERO-CIMS from April and May, 2018. Organic compounds exhibiting similar temporal behavior during isothermal evaporation were grouped into distinct factors. The PMF results were analyzed using the PMF Evaluation Tool (PET v3.05).^42^ Based on the PMF results on the raw particle-phase data, each PMF factor was identified either as a sample or background factor according to its contribution in the particle and filter blank samples. We found a solution with 5 sample factors and 3 background factors (8 factors in total) to represent our data best. The PMF residual for this solution is 6%.

To identify the source regions of the individual PMF sample factors, we correlated their time series with detailed air mass origin and transport pathway information. Aliaga *et al.*^43^ identified source regions of air masses arriving at the measurement site. This analysis relied on source–receptor relationships (SRR) derived from backward WRF-FLEXPART simulations combined with a *k*-means clustering approach. The time series of the PMF factors were correlated to the time series of 18 SRR clusters. The time series of the Amazon factor correlated well with air masses originating from the Amazon region, *i.e.* 4 out of 18 SRR clusters, with correlation coefficients ranging from 0.5 to 0.68. The percentages of air in each of the 4 clusters that had travelled over tropical and subtropical moist broadleaf forests were 84%, 56%, 90% and 89% for the 4 clusters, respectively.

## Conclusions

In this study, we report the direct observations of 2-methyltetrols in both the gas- and particle phases in air masses originating from Amazonia and transported to the high-altitude mountain station on Mount Chacaltaya in the Bolivian Andes at 5240 m a s l, at the edge of the Amazon basin. This compound accounted for 12% of the total particulate mass attributed to the Amazon factor, as determined by PMF analysis of two months of particle-phase measurements. Using a chemical box model coupled with cloud microphysics we show that transport of 2-methyltetrols in the gas phase from the Amazon boundary layer to the upper troposphere through convective systems is likely not the main transport pathway for the 2-methyltetrol we observe, as it may be taken up by hydrometeors in the convective cloud. Isoprene oxidation and reactive uptake of IEPOX inside the cloud systems, or release of 2-methyltetrols in the upper troposphere and outflow upon droplet evaporation or freezing may be pathways for 2-methyltetrols to be transported to the upper troposphere. We then show that transport to the station at lower altitude and thus higher temperature can lead to potential evaporation and our observations of both the gas- and particle-phase 2-methyltetrol.

## Author contributions

Conceptualization: CM, CW, IR, MS, JT, FB, MA; investigation: CW, CM, FB; data curation: CW; formal analysis: CW, AC; funding acquisition: CM, IR, MA, FB; writing: CW, CM, AC.

## Conflicts of interest

There are no conflicts to declare.

## Data Availability

All data are available upon request from the corresponding authors.
